# Improving the Therapeutic Effect of Ultrasound Combined With Microbubbles on Muscular Tumor Xenografts With Appropriate Acoustic Pressure

**DOI:** 10.3389/fphar.2020.01057

**Published:** 2020-07-15

**Authors:** Yan He, Meiling Yu, Jie Wang, Fen Xi, Jiali Zhong, Yuwen Yang, Hai Jin, Jianhua Liu

**Affiliations:** ^1^ Department of Medical Ultrasound, Guangzhou First People’s Hospital, School of Medicine, South China University of Technology, Guangzhou, China; ^2^ Department of Functional Examination, Xiamen Hospital of Traditional Chinese Medicine, Xiamen, China

**Keywords:** ultrasound combined with microbubbles, muscular tumor xenograft, anti-vascular therapy, acoustic pressure, microvessel density

## Abstract

Ultrasound combined with microbubbles (USMB) is a promising antitumor therapy because of its capability to selectively disrupt tumor perfusion. However, the antitumor effects of repeated USMB treatments have yet to be clarified. In this study, we established a VX2 muscular tumor xenograft model in rabbits, and performed USMB treatments at five different peak negative acoustic pressure levels (1.0, 2.0, 3.0, 4.0, or 5.0 MPa) to determine the appropriate acoustic pressure. To investigate whether repeated USMB treatments could improve the antitumor effects, a group of tumor-bearing rabbits was subjected to one USMB treatment per day for three consecutive days for comparison with the single-treatment group. Contrast-enhanced ultrasonic imaging and histological analyses showed that at an acoustic pressure of 4.0 MPa, USMB treatment contributed to substantial cessation of tumor perfusion, resulting in severe damage to the tumor cells and microvessels without causing significant effects on the normal tissue. Further, the percentages of damaged area and apoptotic cells in the tumor were significantly higher, and the tumor growth inhibition effect was more obvious in the multiple-treatment group than in the single USMB treatment group. These findings indicate that with an appropriate acoustic pressure, the USMB treatment can selectively destroy tumor vessels in muscular tumor xenograft models. Moreover, the repeated treatments strategy can significantly improve the antitumor effect. Therefore, our results provide a foundation for the clinical application of USMB to treat solid tumors using a novel therapeutic strategy.

## Introduction

The development of safe and effective antitumor therapies remains a major research hotspot. The use of ultrasound combined with microbubbles (USMB) has recently emerged as a promising method for destroying the tumor vasculature because of its ability to cause complete and persistent cessation of blood flow to the tumor (Wood and Sehgal, 2015; Yang et al., 2016), which is mainly considered to occur through vascular dilation (Wood et al., 2007; Wood et al., 2008; Levenback et al., 2012), thrombosis (Huang et al., 2013; Foiret et al., 2017), and ultimate disruption of blood perfusion to the tumor (Liu et al., 2012; Zhang et al., 2018). Liu et al. (2012) reported that USMB treatment with a peak negative acoustic pressure of 4.8 MPa can block the circulation of subcutaneous tumor xenografts for 24 h, resulting in long-term tumor remission and improved survival. Moreover, diffuse hematoma accompanied by thrombosis and intercellular edema were detected in response to extensive damage to the tumor microvessels. Accumulating evidence has also demonstrated that the persistent cessation of tumor perfusion during USMB therapy is associated with a significant decrease in microvessel density (MVD), indicating that the substantial destruction of endothelial cells and resultant vascular depletion can cause permanent shutdown of the tumor blood flow (Eichhorn et al., 2010; Abma et al., 2019).

Tumor neovessels have been suggested to be more prone to destruction by USMB therapy because of their defective construction. Particularly, most peripheral cells of tumor neovessels show an abnormal morphology, and cytoplasm protrusions extend in reverse (away from the vessel walls) and loosely surround endothelial cells (Morikawa et al., 2002). Higher permeability was observed in response to discontinuous endothelial cells with rare tight junctions between the endothelial cells (Ribatti et al., 2007). Wang et al. (2015) observed large-scale necrosis and apoptosis of tumor cells after USMB therapy at an acoustic pressure of 3.0 MPa, with no significant effects on the surrounding muscles and skin tissue.

Acoustic cavitation, particularly inertial cavitation, plays an essential role in the anti-vascular effect elicited by USMB treatment (Lin et al., 2012; Liu et al., 2012), and acoustic pressure is among the most important parameters for inertial cavitation (Abbott, 1999; Tu et al., 2018). Based on these previous findings, when using an appropriate pressure, USMB therapy can result in substantial and persistent damage to tumor perfusion without damaging the surrounding normal tissue. However, most studies in this field have focused on the effect of a single treatment on a subcutaneous tumor model. Few studies have investigated whether the antitumor effect is enhanced by increasing the number of treatments.

Therefore, this study was conducted to establish a muscular tumor xenograft model and determine whether USMB treatment with an appropriate acoustic pressure can achieve substantial blockage of tumor blood perfusion without causing significant damage to the normal tissue. Further, to investigate whether repeated USMB treatments could improve the antitumor effects, two USMB treatment strategies (repeated and single) were applied. These data will contribute to improving the clinical application of USMB therapy to directly ablate solid tumors.

## Materials and Methods

### Ethics Statement

The study was approved by the Animal Research Committee of the Guangzhou First People’s Hospital, South China University of Technology, and was performed in accordance with the NIH guide for the Care and Use of Laboratory Animals.

### Animals and Model Establishment

84 male New Zealand white rabbits (2.0–2.2 kg, 120–150 d) were obtained from the Animal Center of Guangdong Medical Laboratory (Guangzhou, China). Before inoculation, all rabbits were reared for at least 7 days at 24°C to 26°C under 45% to 55% humidity. VX2 tumor tissue specimens were purchased from the cell bank of Sun Yat-sen University (Guangzhou, China). The tumor tissues were cut into small pieces (1 mm3) and placed in a culture dish with physiological saline solution, and then injected into the muscle layer of the rabbit right hind limb (2.6 ± 0.5 mm from the surface) (Zhang et al., 2019). The USMB treatment was performed when the tumor reached a length of 10.0 ± 0.7 mm and width of 5.0 ± 0.3 mm.

### Microbubbles and Pulsed Therapeutic Ultrasound Device

**Figure 1:**
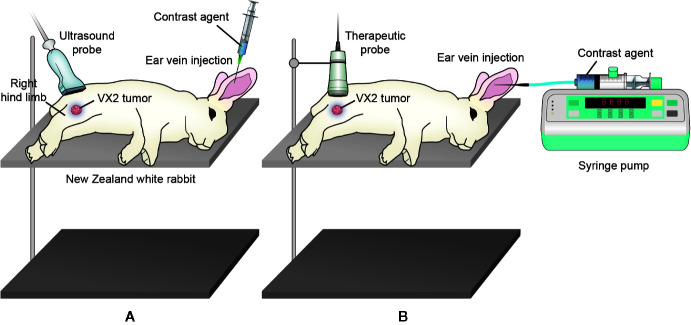
Illustration of B-mode and CEUS imaging and USMB treatment. **(A)** B-mode imaging was firstly performed. After intravenous bolus injection of microbubbles, CEUS imaging was initiated. **(B)** For USMB treatment, the microbubbles suspension was injected into the ear vain of rabbits at a uniform rate of 1.25 ml/min *via* a micro syringe pump.

Zhifuxian (Wei et al., 2019), a second-generation microbubble contrast agent, was developed by the Department of Ultrasound, Xinqiao Hospital, The Third Military Medical University, Chongqing, China. In brief, a suspension of dipalmitoyl phosphatidylglycerol (DPPG), distearoyl phoshatidylcholine (DSPC), and polyethylene glycol-4000 (PEG-4000) was lyophilized and then agitated with perfluoropropane gas using a high-speed mechanical amalgamator (Liu et al., 2011). The mean microbubble diameter was approximately 2 µm, and the concentration was 6 to 9 × 10^9^/ml. The doses of Zhifuxian for contrast-enhanced ultrasound (CEUS) imaging and USMB therapy were 0.01 and 0.1 ml/kg, respectively. The microbubbles were shaken for 30 s before injection. After intravenous injection of a bolus of 1.5 × 10^7^ microbubbles, CEUS imaging was initiated simultaneously with 2-ml saline injection (**Figure 1A**). For USMB treatment, 1.5 × 10^8^ microbubbles were firstly added to 5 ml normal saline and gently shaken to prepare a homogeneous suspension. The suspension was injected into the ear vain of rabbits at a uniform rate of 1.25 ml/min *via* a micro syringe pump (LD-P2020, Shanghai Lande Medical Equipment Co. Ltd., Shanghai, China) within 4 min (**Figure 1B**).

USMB treatments were performed using a pulsed therapeutic ultrasound device with a KHT-017 transducer (DCT-700, Shenzhen Well. D Medical Electronics Co. Ltd., Shenzhen, China). The transducer was driven by a wave generator and a specially designed power amplifier (Mianyang Sonic Electronic Ltd., Mianyang, China), which was developed by Liu et al. (2012). The transducer was composed of an aluminum shell with the tip covered by a polyimide membrane. The effective therapeutic diameter was 2 cm. During the treatment process, the transducer was fixed on a cross steel stand. A needle hydrophone (TNU001A, NTR, Seattle, WA, USA) was positioned to measure the acoustic output at a depth of 0.5 cm from the surface. A non-focused ultrasound was used with the following acoustic parameters: frequency of transducer = 1.0 MHz, acoustic pressure = 1.0–5.0 MPa, pulse repetition frequency = 10 Hz, intermittent mode of transducer = 9 s (on) and 3 s (off), duty cycle = 0.2%, insonation time = 5 min (Zhang et al., 2019). Treatment was initiated at least 15 min after the previous CEUS imaging to ensure the clearance of all contrast agents. During the USMB treatment, 5 ml prepared microbubble suspension was injected uniformly at a rate of 1.25 ml/min within 4 min using a syringe pump (**Figure 1B**). Subsequently, an intravenous drip of normal saline was administered to fully circulate the microbubbles within the remaining 1 min.

### Experimental Protocol

To determine the appropriate level of ultrasound pressure, 48 tumor-bearing rabbits were randomly divided into eight groups (n = 6 per group), including one microbubbles only (MBs) group, five USMB groups, and two ultrasound-only (US) groups. Rabbits in the MBs group were injected intravenously with microbubbles in the absence of ultrasound, and those in the USMB groups were insonated with different peak negative acoustic pressure levels (1.0, 2.0, 3.0, 4.0, or 5.0 MPa) in the presence of microbubbles. In the US group, the rabbits were insonated with a peak negative acoustic pressure of 4.0 MPa or 5.0 MPa in the presence of normal saline rather than the microbubble suspension. All rabbits were anesthetized during the experiments by intramuscular injection of 2% sodium pentobarbital (20 mg/kg) compounded with xylazine hydrochloride (0.15 ml/kg). The hair on the right hind limb was shaved to create an acoustic window for ultrasound imaging. Serial B-mode and CEUS imaging of the tumor were performed before and immediately after insonation. After the rabbits were sacrificed, tumors, thigh muscles, and skin tissue were collected for hematoxylin and eosin (H&E) and immunohistochemical (IHC) staining.

To observe whether multiple USMB treatments could enhance the antitumor effect, 18 tumor-bearing rabbits were randomly divided into a control group, single USMB treatment group, and multiple USMB treatments group (n = 6 per group). The control group was treated with neither ultrasound treatment nor microbubble injection. The single USMB treatment group was insonated with microbubbles only once, whereas the multiple USMB treatments group was insonated with microbubbles once per day for three consecutive days. Serial B-mode and CEUS imaging of the tumor were performed before and immediately after insonation. After the rabbits were sacrificed, the tumors, thigh muscles, and skin samples were collected for H&E and terminal deoxynucleotidyl transferase dUTP nick-end labeling (TUNEL) staining.

To investigate the tumor remission effect of various USMB strategies, another 18 tumor-bearing rabbits were also randomly divided into a control group, single USMB treatment group, and multiple USMB treatment group (n = 6 per group). The volume of tumors was measured at days 1, 5, 10, and 15 after the treatment. The experimental protocol is also illustrated in supplementary data.

### B-mode and CEUS Perfusion Imaging

B-mode and CEUS perfusion imaging were performed using an ultrasound system with an L12-5 transducer (Philips IU22; Royal Dutch Philips Electronics Ltd., Amsterdam, the Netherlands). Rabbits were anesthetized and placed in the lateral position. Initial B-mode imaging was performed at a mechanical index of 0.27. Next, CEUS imaging was performed at a depth of 3 cm and a mechanical index of 0.07. The dynamic image of the subsequent 1 min was obtained. The probe was kept stationary to maintain the tumor in a fixed position during the imaging process (**Figure 1A**).

The peak intensity (PI) parameter was used to assess the extent of blood perfusion in the tumors. Two doctors with vast CEUS experience obtained the PI values using Philips Qlab^®^ software (Version 8.1.2, Philips Medical Systems, Andover, MA, USA) in offline analysis. The entire tumor was selected as the region of interest and any non-tumor tissue was excluded to the greatest extent possible. The software then automatically plotted the time-intensity curve and calculated the PI simultaneously.

The tumor volume was evaluated using calipers and B-mode ultrasound results. The length (L) and width (W) of the tumor were measured with an L12-5 transducer at a mechanical index of 0.6, and the tumor volume (V) was calculated as follows: π × (L × W^2^)/6 (Leite De Oliveira et al., 2012).

### H&E Staining

The tumors, thigh muscles, and skin specimens were fixed with 4% paraformaldehyde, paraffin-embedded, and cut into 4 µm slices for H&E staining. Damage to the tumors (the vasculature and the cells) was identified and quantified independently by two experienced pathologists using an optical microscope (Axio Scope A1, ZEISS, Oberkochen, Germany). The percentage of the damaged area, defined as the area of damage per field of view divided by the total area of the field of view, was calculated using Image Pro Plus 6.0 software (Media Cybernetics, Silver Spring, MD, USA). Ten 400-fold magnified fields were randomly selected to calculate the damaged area and injured vessel count, and the mean value was used as the quantified degree of tumor damage and vascular injury.

### IHC

To evaluate the MVD, the slices were incubated with anti-CD31 antibodies (1:25, Abcam, Cambridge, UK) to stain the endothelial cells. MVD counting was performed by two experienced pathologists independently as described previously (Weidner et al., 1991; Jacquemier et al., 1998). Ten 400-fold magnified fields were randomly selected to count the MVD, and the mean value was taken as the MVD value of the slice.

### Apoptosis Assay

To determine the apoptosis level, a TUNEL assay was performed using an *in situ* cell death detection kit (POD, Roche, Germany) as previously described (Zeng et al., 2014). The TUNEL-labeled cells were defined as positive cells. The mean percentage of TUNEL-labeled cells was calculated from 10 randomly selected fields under an epifluorescence microscope (Axio Scope A1, ZEISS, Oberkochen, Germany).

### Statistical Analysis

Multiple comparisons were performed by one-way analysis of variance (ANOVA) followed by a Bonferroni correction. The least-squares method was employed to assess linear correlations between selected variables. Comparisons of tumor volume among groups were performed using repeated-measures ANOVA. The results are expressed as the mean ± standard deviation unless otherwise specified and *p* < 0.05 was considered as statistically significant. All statistical analyses were performed using SPSS 13.0 software (IBM SPSS, Chicago, IL, USA).

## Results

### Effects of USMB Treatment with Varying Pressure on the Tumor, Muscle, and Skin

#### CEUS Imaging

**Figure 2:**
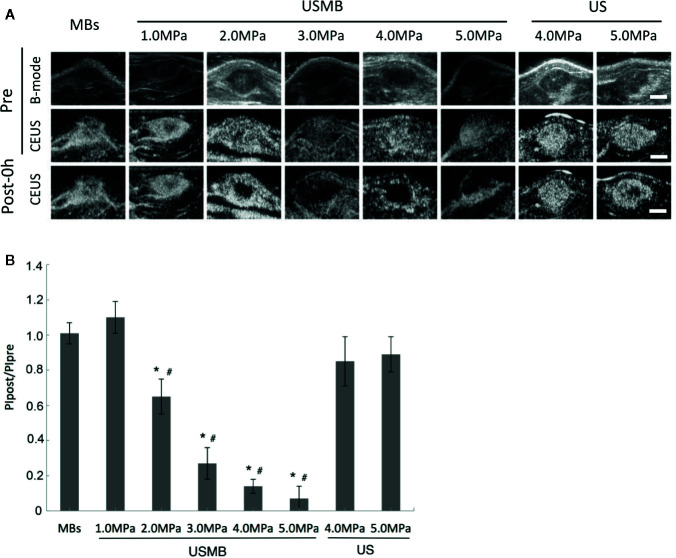
Effects of USMB treatment on tumor blood perfusion with various levels of acoustic pressure. **(A)** Representative B-mode and CEUS images of the tumor from one animal in each group. Scale bar = 0.5 cm. **(B)** Quantitative analysis of the change in tumor blood perfusion. *p < 0.05 vs. PIpre, #*p* < 0.05 vs. the MBs group. PI, peak intensity.

CEUS imaging indicated that the tumors were quickly and completely filled with microbubbles in all groups before treatment. There was no significant reduction in tumor blood perfusion immediately after treatment in the MBs and USMB groups at an acoustic pressure of 1.0 MPa (**Figures 2A, B**). However, gradual reduction in blood perfusion in the tumor was observed with increased acoustic pressure at 2.0, 3.0, 4.0, and 5.0 MPa (**Figures 2A, B**). In the groups treated with USMB at 3.0 or 4.0 MPa, blood perfusion was completely blocked in the center of the tumor, but rim enhancement was still visible at the edges. However, blood perfusion was also interrupted in the muscle surrounding the tumor in the 5.0-MPa USMB group (**Figure 2A**). There was no obvious change in tumor blood perfusion in the 4.0-MPa US treatment group, whereas 5.0-MPa US treatment resulted in a slight decrease of blood perfusion in the center of the tumor (**Figure 2A**) although the difference was not significant (**Figure 2B**).

#### H&E Staining

**Figure 3:**
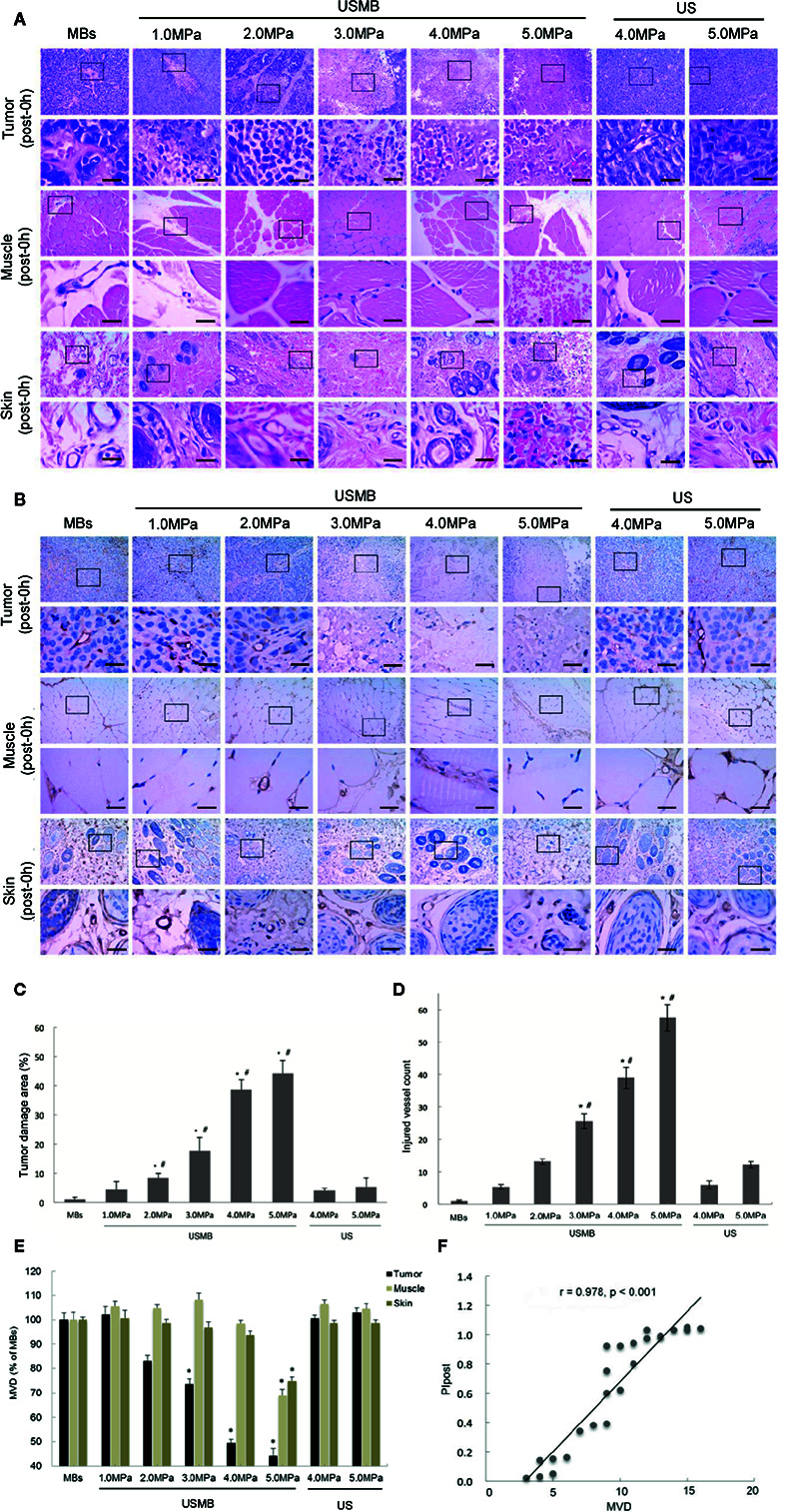
Effects of USMB treatment with various levels of acoustic pressure on cells and microvessels in the tumor, muscle, and skin. Representative images of hematoxylin-eosin **(A)** and immunohistochemical **(B)** staining. Scale bar = 20 µm. Quantitative analysis of the damaged area **(C)**, injured vessel count in the tumor **(D)**, and MVD in the tumor, muscle, and skin **(E)**. **(F)** Correlations between MVD and PI in the tumor immediately after treatment. *p <0.05 vs. MBs group, ^#^ *p* <0.05 vs. all treatment groups. MVD, microvessel density. PI, peak intensity.

H&E staining clearly showed that tumor cells and tumor microvessels were intact in the MBs group, and no extravasated red blood cells (RBCs) were observed immediately after treatment (**Figure 3A**). In the USMB treatment groups, higher acoustic pressure was accompanied by more severe damage to the tumor cells and tumor microvessels. More extensive extravasation of RBCs into the interstitial space was also found with increased acoustic pressure. In the 1.0- and 2.0-MPa USMB treatment groups, only scattered degenerative changes in tumor cells and focal RBCs extravasation were observed (**Figure 3A**). However, the structures of tumor cells and tumor microvessels were severely disrupted in the 4.0- and 5.0-MPa USMB groups immediately after treatment. Most microvasculature structures could not be identified and a large area of hemorrhage was observed. The degree of injury was moderate in the 3.0-MPa USMB group (**Figure 3A**). In contrast to the tumor findings, significant hemorrhage in the muscle or skin was only observed in the 5.0-MPa USMB group immediately after treatment. There was no obvious extravasation of RBCs or cell necrosis in the tumor, muscle, or skin of the US treatment groups at either 4.0 or 5.0 MPa (**Figure 3A**).

#### IHC

IHC findings further confirmed that the structures of the microvessels in the tumor, muscle, and skin were retained in the MBs group immediately after treatment (**Figure 3B**). In the 1.0- and 2.0-MPa USMB treatment groups, most tumor microvessels were complete, although scattered vessel fragments were detected immediately after treatment. Severely ruptured microvessels and microvascular debris were observed in the 4.0- and 5.0-MPa USMB treatment groups. In some injured areas, even the vascular fragments were indistinguishable (**Figure 3B**). In the 3.0-MPa USMB treatment group, the degree of tumor microvessels injury was intermediate. In contrast, ruptured microvessels in the muscle and skin were only observed in the 5.0-MPa USMB group immediately after treatment. No obvious microvessel rupture was observed in the tumor, muscle, or skin of the two US groups immediately after treatment (**Figure 3B**). Compared to the MBs group, there was a significant reduction in the tumor MVD in the 3.0-, 4.0-, and 5.0-MPa USMB treatment groups; the reduction was more obvious with increasing acoustic pressure (**Figure 3B**). However, no significant reduction in the MVD of the tumor was observed in the 1.0- and 2.0-MPa USMB treatment groups (**Figure 3B**).

Quantitative analysis of the tumor damage area and vascular injury degree also revealed that the damage to the tumor gradually increased with increasing acoustic pressure in the USMB treatment groups (**Figures 3C, D**). In the 3.0-, 4.0-, and 5.0-MPa USMB treatment groups, the MVD of the tumor was decreased by 31.03%, 47.78% and 55.96%, respectively (*p* < 0.05). However, significant reductions in the MVD of the muscle and skin (31.03% and 25.03%, respectively) were only observed in the 5.0-MPa USMB treatment group. In the two US groups, no significant difference in the MVD of the tumor, muscle, or skin was found compared to that of the MBs group (**Figure 3E**). In addition, a significant correlation between the MVD and PI in the tumor immediately after USMB treatment was observed (*p* < 0.05; **Figure 3F**).

### Antitumor Efficacy of Single and Multiple USMB Treatments

#### CEUS Imaging

**Figure 4:**
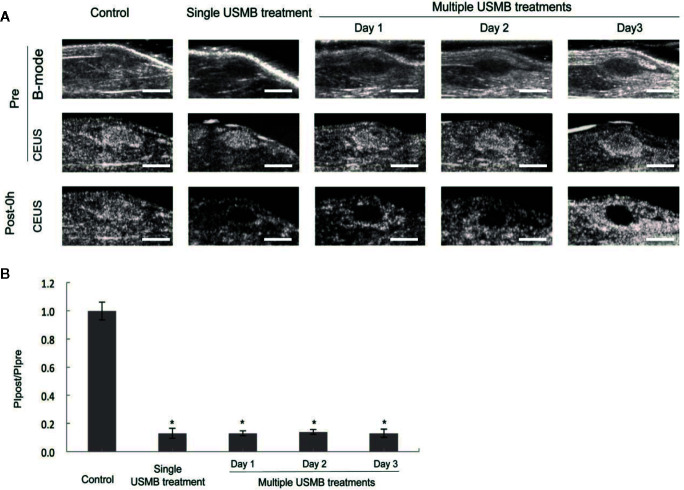
Anti-vascular effect (cessation of tumor blood perfusion) caused by different USMB treatment strategies (single or repeated) with an acoustic pressure of 4.0 MPa. **(A)** Representative B-mode and CEUS images of the tumor from one animal in each group. Scale bar = 1 cm. **(B)** Quantitative analysis of the change in tumor blood perfusion. *p < 0.05 vs. control group. PI, peak intensity.

In the 4.0-MPa USMB treatment group, a significant antitumor effect was observed with no significant damage to the muscle or skin. Therefore, 4.0 MPa was considered to be the appropriate acoustic pressure for treating muscular tumor xenografts and was applied in subsequent experiments. As shown in **Figure 4A**, there was no significant difference in blood perfusion in tumors from all groups prior to treatment. However, immediately after treatment, tumor blood perfusion was nearly completely blocked in both the single and multiple USMB treatment groups (*p* < 0.05), with no significant difference in the degree of blood blockage in the tumor between the two groups (**Figures 4A, B**). In the multiple treatments group, tumor perfusion was recovered before each repeated treatment (**Figure 4A**).

#### H&E Staining

**Figure 5:**
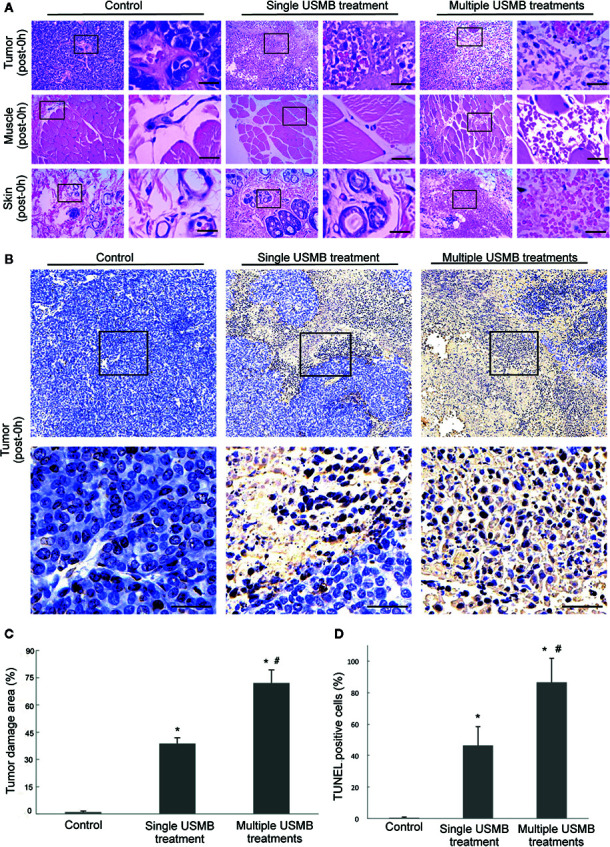
Damage to cells and microvessels in the tumor, muscle, and skin caused by different USMB treatment strategies. **(A)** Representative images of hematoxylin-eosin staining. **(B)** TUNEL-labeled apoptotic cells in the tumor. Scale bar = 20 µm. Quantitative analysis of the tumor damage area **(C)** and TUNEL-labeled cells in the tumor **(D)**. *p < 0.05, vs. control group; ^#^ *p* < 0.05, vs. all treatment groups.

Nuclear fragmentation, nuclear dissolution, and even complete destruction of tumor cell structures were observed by H&E staining immediately after treatment in both the single and multiple treatment groups (**Figure 5A**). In addition, severe damage to the microvasculature was observed in both groups, although the degree of tumor cell damage and RBCs leakage was more severe in the multiple treatments group. In contrast to the effects on the tumors, no RBCs leakage was found in the muscle or skin tissues in the single treatment group but was detected in the multiple treatments group (**Figure 5A**). This was confirmed by visual observation of subcutaneous bleeding immediately after the third treatment. The percentage of tumor damage area was increased by 38.69% and 72.04% in the single and multiple treatment groups, respectively (*p* < 0.05), with a more significant increase in the latter group (*p* < 0.05) (**Figure 5C**).

#### Apoptosis Assay

The TUNEL assay revealed that the percentage of tumor cell apoptosis increased significantly in the USMB treatment groups as compared to the control group (*p* < 0.05, **Figures 5B, D**). Further, there was a significantly greater increase of 75.91% apoptotic cells in the multiple treatments group compared to the 45.09% increase in apoptosis in the single treatment group (*p* < 0.05, **Figure 5D**).

#### Tumor Volume Measurement

**Figure 6:**
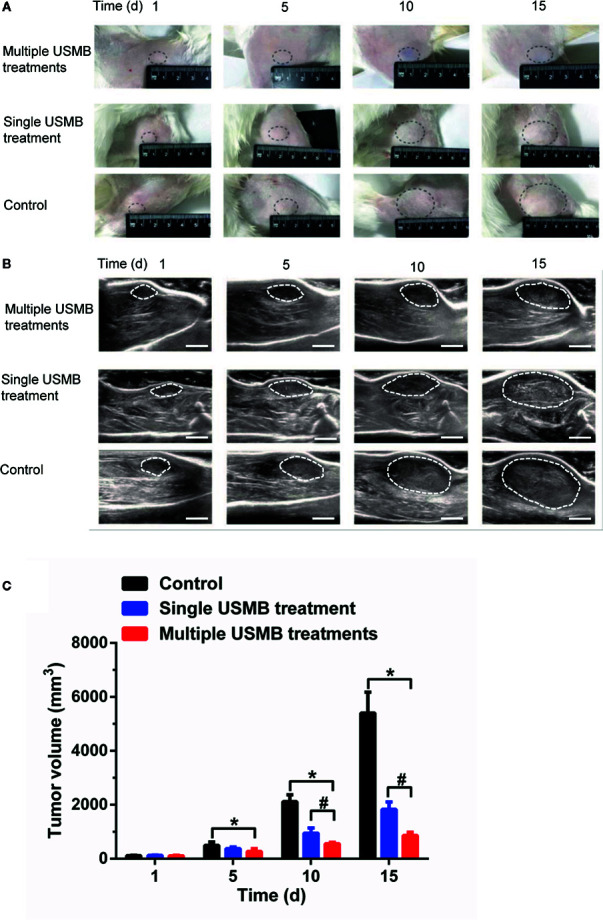
Tumor growth suppression effect of different USMB treatment strategies. Representative images **(A)** and B-mode ultrasound images **(B)** of the tumor from one animal in each group. Scale bar = 1 cm. **(C)** Quantitative analysis of the tumor volume. *p < \0.05, vs. control group; ^#^ *p* < 0.05, vs. all treatment groups.

Before treatment, there was no significant difference in tumor volume between the treatment and control groups (**Figures 6A–C**). The tumor volume in the single treatment group was significantly smaller than that in the control group at days 10 and 15 after treatment, whereas the tumor volume of the multiple treatment group was the lowest at each measurement time point after treatments (all *p* < 0.05, **Figure 6C**).

## Discussion

In this study, we established an *in vivo* model to determine the most appropriate acoustic pressure for treating muscular tumor xenografts in New Zealand rabbits. We found that USMB treatment with an acoustic pressure of 4.0 MPa resulted in substantial damage to tumor perfusion but had no significant effect on the muscle and skin. In the USMB treatment groups with acoustic pressures of 1.0, 2.0, 3.0, and 5.0 MPa, there was either incomplete cessation of tumor perfusion or an obvious reduction in the blood perfusion of normal tissues, whereas no significant change in tumor perfusion was observed in the US treatment groups. These findings suggest that with an acoustic pressure of 4.0 MPa at a frequency of 1.0 MHz, USMB treatment effectively stopped blood flow in a muscular tumor xenograft without reducing blood perfusion in the normal tissues. This observation provides further evidence for the clinical use of this emerging anti-vascular therapy. Moreover, compared to previous studies performed in subcutaneous xenograft tumors (Liu et al., 2012; Wang et al., 2015), an ultrasound pressure of 4.0 MPa is in the intermediate range, whereas the frequency we employed was slightly higher than those used previously (0.831 and 0.94 MHz, respectively).

The large amount of RBC leakage from the damaged vessels in this study confirmed that USMB treatment led to vessel injury or even rupture. Moreover, the compression effects from intercellular edema and hematoma formation (Bunte et al., 2006; Liu et al., 2012) along with platelet activation (Hu et al., 2012) can further reduce tumor blood perfusion. A reduction in MVD and a close correlation between the MVD and lasting tumor blood perfusion were also observed in the present study. Accordingly, the substantial depletion of tumor microvessels at least partly contributed to the sustained cessation of tumor blood flow.

This study demonstrated that tumor vessels were more vulnerable to destruction by USMB treatment than the vessels of normal tissues, which is consistent with the results of previous studies (Liu et al., 2012; Wang et al., 2015). Normal vessels typically evolve with an intact and strong wall structure, including integrated endothelial cells, basement membrane, and smooth muscle layers (Liu et al., 2012). Although USMB can injure the vascular endothelia, the damage is limited to endothelial malformation or contraction of the endothelial cells without leading to a flow obstruction (Hwang et al., 2006). Therefore, vessels of normal tissues with a robust wall can resist mechanical cavitation damage from USMB treatment and survive. However, most tumor vessels are immature and defective. The connections between endothelial cells of tumor vessels are incompact because of the lack of tight junctions (Ribatti et al., 2007); the pericytes show multiple abnormalities and are loosely associated with endothelial cells (Morikawa et al., 2002). Furthermore, despite the extensive vessel coverage, the basement membrane has a loose association with endothelial cells and pericytes and extends away from the vessel wall (Baluk et al., 2003). Thus, USMB treatment takes advantage of tumor neovasculature as a vulnerable target and can be used as a potential physical antitumor method.

To explore whether repeated USMB treatments with an appropriate ultrasound pressure could improve the antitumor effects compared to a single treatment, we established a multiple USMB treatment group in which USMB treatment with an acoustic pressure of 4.0 MPa was administered once a day for three consecutive days. The percentages of tumor damage and apoptotic tumor cells obviously increased in the multiple treatment group. Moreover, tumor growth was delayed to a greater extent. These results suggest that compared to a single USMB treatment, antitumor efficacy was enhanced by increasing the number of treatments, which is consistent with the results of Duvshani-Eshet et al. (2007). However, although the therapeutic effect was superior after multiple USMB treatments compared to a single treatment, RBC leakage in the muscles and skin was also more extensive compared to that in the single-treatment group. Fortunately, this is a mild and reversible adverse reaction.

USMB treatment is generally considered to delay tumor growth by disrupting tumor blood perfusion and causing tumor cell necrosis. Wu and Nyborg (2008) found that the inertial cavitation caused by microbubbles not only destroyed tumor microvessels but also resulted in direct tumor cell damage. Moreover, Zhang et al. (2014) suggested that the downregulation of vascular endothelial growth factor receptor-2 and αvβ3 integrin expression was partly responsible for the suppression of tumor growth with ultrasound treatment. In this study, although no significant decrease in blood perfusion in the tumor was observed after multiple treatments, the percentage of apoptotic tumor cells was obviously increased compared to that in the single-treatment group. Tumor growth was also delayed to a greater extent in the repeated USMB treatment group. These results suggest that in addition to blocking the tumor blood flow, other factors cause tumor cell apoptosis and thereby inhibit tumor growth during repeated USMB treatments. However, the specific mechanism remains unclear.

Despite the significant obstruction of circulation caused by 4.0-MPa USMB treatment in muscular tumor xenografts, complete cessation of the tumor circulation persisted for less than 24 h. However, a previous study showed that 4.8-MPa USMB treatment completely blocked blood perfusion of subcutaneous Walker 256 tumors in Sprague–Dawley rats for 24 h (Liu et al., 2012). In this study, however, we found that an acoustic pressure of 4.0 MPa was not high enough to block the blood flow to muscular tumor xenografts for 24 h or more. We hypothesized that the vessels in muscular tumor xenografts were not completely ruined during the 4.0 Mpa USMB treatment. Therefore, as the compression effects from intercellular edema and hematoma formation gradually diminished, blood perfusion gradually recovered.

There are several limitations to this study that should be considered. First, although the muscular tumor xenograft model we built in this study was deeper than a subcutaneous tumor model, it was still relatively superficial compared to most clinical tumors. The appropriate parameters and the antitumor effects of USMB treatment on deep tumor models are still uncertain. Therefore, a VX2 New Zealand rabbit liver tumor model should be built in future studies to preferably explore the therapeutic potential of USMB treatment on clinical tumors.

Second, although the TUNEL assay revealed that the apoptosis cells increased in the USMB treatment groups, the cell types (endothelial cells or tumor cells) can be further differentiated by counterstaining.

Finally, the recovery of tumor blood perfusion within 24 h may create an obstacle for the clinical application of USMB treatment. However, given the security of multiple USMB treatments, the treatment interval should be shortened rather than increasing the therapeutic sound pressure in future studies. We will increase the number of observation time points to determine the recovery time of tumor blood perfusion, and thus conclusively determine the treatment interval. The optimal frequency of repeated USMB treatments should be investigated, and its combined use with other treatments such as chemotherapy or radiotherapy should also be tested in future studies.

## Data Availability Statement

All datasets generated for this study are included in the article/**Supplementary Material**.

## Ethics Statement

The animal study was reviewed and approved by Animal Research Committee of Guangzhou First People’s Hospital.

## Author Contributions

YH, MY, and JL conceived and designed the experiments and wrote the paper. YH and MY performed the experiments and analyzed the data. JW, FX, JZ, YY, and HJ contributed reagents/materials/analysis tools. All authors contributed to the article and approved the submitted version.

## Funding

This work was supported by the Science and Technology Planning Project of Guangdong Province, China (No. 20130319c) and the Key Clinical Technology Program of Guangzhou, China (2019TS49).

## Conflict of Interest

The authors declare that the research was conducted in the absence of any commercial or financial relationships that could be construed as a potential conflict of interest.
