# Supplementary material for: Improving the Therapeutic Effect of Ultrasound Combined With Microbubbles on Muscular Tumor Xenografts With Appropriate Acoustic Pressure
Source: Front Pharmacol. 2020 Jul 15;11:1057. doi: 10.3389/fphar.2020.01057 (PMC7373785; doi:10.3389/fphar.2020.01057)
Supplement: Supplementary file 1 [file DataSheet_1.pdf]

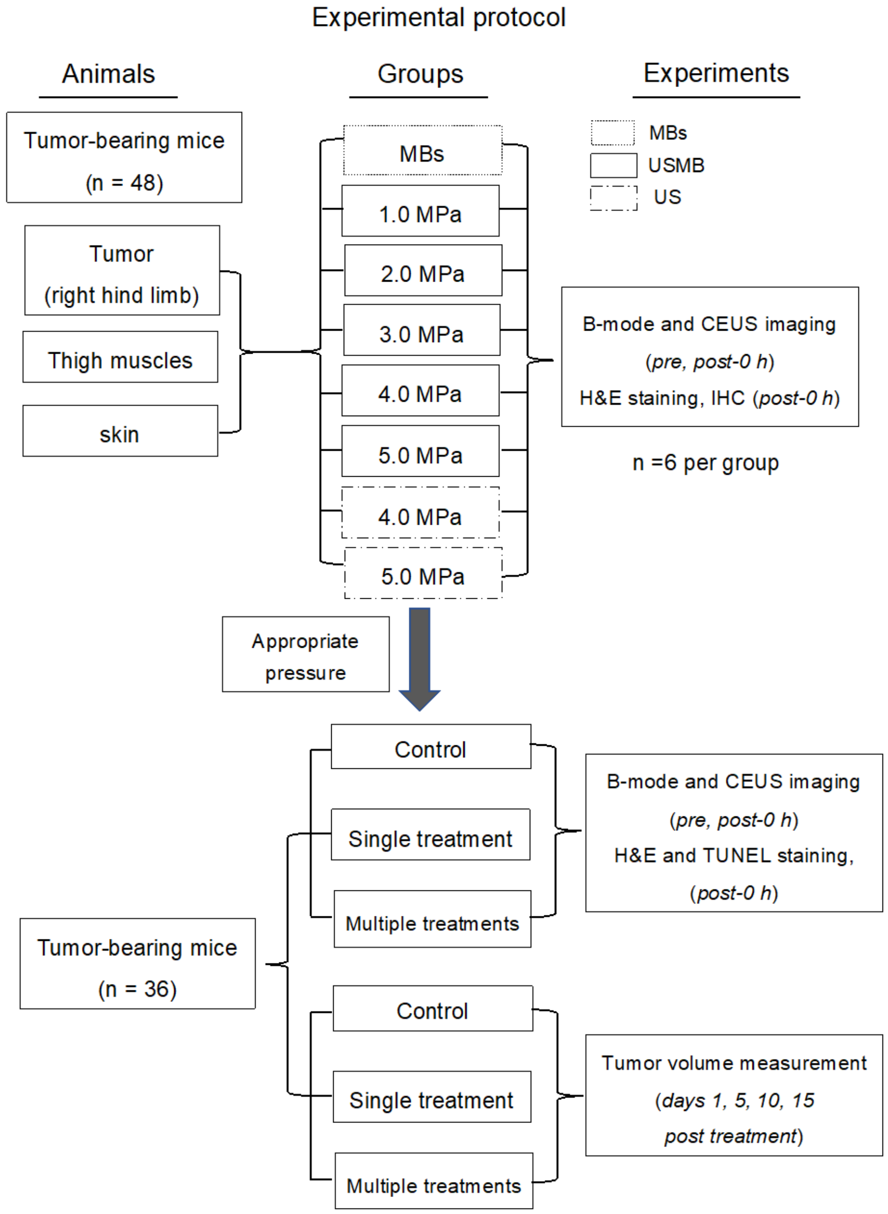

Figure S1. Illustration of the experimental protocol. H&E, hematoxylin and eosin. IHC, immunohistochemical. TUNEL, erminal deoxynucleotidyl transferase dUTP nick-end labeling.
